# Phytolith assemblages reflect variability in human land use and the modern environment

**DOI:** 10.1007/s00334-023-00932-2

**Published:** 2023-06-27

**Authors:** Nina H. Witteveen, Cheryl White, Barbara A. Sanchez Martinez, Roemer Booij, Annemarie Philip, William D. Gosling, Mark B. Bush, Crystal N. H. McMichael

**Affiliations:** 1 Department of Ecosystem and Landscape Dynamics, Institute for Biodiversity and Ecosystem Dynamics, University of Amsterdam, Science Park 904, 1098 GE Amsterdam, Netherlands; 2 Department of History, Faculty of Humanities, Anton de Kom University, Universiteitscomplex, Gebouw 7, Leysweg 86, Paramaribo, Suriname; 3 Institute for Global Ecology, Florida Institute of Technology, Melbourne, FL 32901 USA

**Keywords:** Amazonia, UAV imagery, Human–environment interactions, Suriname, Palaeoecology, Phytoliths

## Abstract

Phytoliths preserved in soils and sediments can be used to provide unique insights into past vegetation dynamics in response to human and climate change. Phytoliths can reconstruct local vegetation in terrestrial soils where pollen grains typically decay, providing a range of markers (or lack thereof) that document past human activities. The ca. 6 million km^2^ of Amazonian forests have relatively few baseline datasets documenting changes in phytolith representation across gradients of human disturbances. Here we show that phytolith assemblages vary on local scales across a gradient of (modern) human disturbance in tropical rainforests of Suriname. Detrended correspondence analysis showed that the phytolith assemblages found in managed landscapes (shifting cultivation and a garden), unmanaged forests, and abandoned reforesting sites were clearly distinguishable from intact forests and from each other. Our results highlight the sensitivity and potential of phytoliths to be used in reconstructing successional trajectories after site usage and abandonment. Percentages of specific phytolith morphotypes were also positively correlated with local palm abundances derived from UAV data, and with biomass estimated from MODIS satellite imagery. This baseline dataset provides an index of likely changes that can be observed at other sites that indicate past human activities and long-term forest recovery in Amazonia.

**Supplementary Information:**

The online version contains supplementary material available at 10.1007/s00334-023-00932-2.

## Introduction

People have lived in Amazonia throughout the Holocene (Roosevelt et al. 1996; Roosevelt 1999). Past human activities such as forest burning, deforestation, soil amendments, raised field agriculture, and shifting cultivation may have left ecological legacies that persist in the modern landscape (Denevan 2001; Lehmann et al. 2003; Levis et al. 2017; Maezumi et al. 2018; McMichael 2021). For example, the modern abundance of palms such as *Euterpe* in the Brazilian Amazon may be due to past palm enrichment (Smith 2015). Successional trajectories following human influence can vary depending on the timing, intensity, and frequency of past disturbances (Åkesson et al. 2021; McMichael 2021). Forest recovery from past human influences, or disturbances, can take centuries because of the long lifespan of trees (Poorter et al. 2016, 2021). Ecosystem functions such as carbon uptake and storage change over the course of succession (Bauters et al. 2019; Rodrigues et al. 2023). Therefore, measuring the long-term ecological dynamics in Amazon systems is crucial to understanding how forests will respond to current and future pressures.

Past vegetation changes following (human) disturbances can be revealed using palaeoecological reconstructions (Bush et al. 2007; Kelly et al. 2018; Loughlin et al. 2018; Duncan et al. 2021). Phytoliths are non-organic silica microfossils formed inside many plants that can be preserved on geological timescales, even in settings where organic matter (including pollen) is not preserved (Piperno 2006; Strömberg et al. 2007). Phytoliths are deposited into terrestrial soils beneath parental plants and are believed to provide a local vegetation signal (Piperno 1988; Crifò and Strömberg 2020). Phytoliths are a suitable proxy for detecting human disturbances in tropical forests, because taxa associated with human land use, such as Poaceae, Arecaceae, and Heliconiaceae, are abundant phytolith producers (Piperno and Becker 1996; Iriarte 2003; Piperno 2006; Chen and Smith 2013; McMichael et al. 2015; Piperno et al. 2019).

To date, phytolith analysis in Amazonia has been relatively qualitative or descriptive in its reconstructions of past human activity and successional trajectories (Åkesson et al. 2021; McMichael 2021; Piperno et al. 2021). Quantitative reconstructions have been developed for palaeoecological proxies in temperate regions, and typically involve generalized linear or additive models, or models based on dissimilarity metrics from ordinations, such as modern analog matching (Bennion et al. 2004; Birks et al. 2012; Birks 2014; Simpson 2018). These models are generally referred to as palaeoecological transfer functions. To link microfossil assemblages to a range of environmental conditions, baseline datasets of microfossils collected a modern environmental gradients are needed. A small number of quantitative studies on phytoliths have shown the potential for reconstructing: (i) past climatic conditions, and (ii) tree cover density in a savanna-forest transition (Lu et al. 2006, 2007; Bremond et al. 2008; An et al. 2015; Biswas et al. 2016; Liu et al. 2018; Li et al. 2022). Although phytoliths have been characterized a several forest types in Amazonia (Dickau et al. 2013; Watling et al. 2020), baseline data of the variance in phytolith representation across gradients of disturbance have not been fully documented. Such data are needed to quantify past human disturbances (such as deforestation) and potential long-term effects on vegetation.

Palaeoecological reconstructions based on microfossil transfer functions are hindered by the lack of a one-to-one relationship between the abundances of microfossils found in a sample and the abundances of that plant in the surrounding environment (Bush and Rivera 1998). With phytolith assemblages, palms, understory herbs and grasses tend to be overrepresented, whereas several woody taxa produce non-diagnostic or no phytoliths (Piperno 2006; Collura and Neumann 2017; Piperno and McMichael 2020; Watling et al. 2020). Several metrics have been derived to distinguish forest types in tropical systems, including the ratio of tree to grass phytoliths (D/P index, hereafter openness index) (Alexandre et al. 1997; Strömberg 2004; Dickau et al. 2013; Astudillo 2018; Testé et al. 2020; Crifò and Strömberg 2021), but these metrics have not been used to differentiate phytolith assemblages across various types of land use.

The tropical rainforests in Suriname contain some of the highest biomass values in Amazonia (Saatchi et al. 2011; Avitabile et al. 2016). Past human activities and their impact on the vegetation (i.e. ecological legacies), however, remain relatively understudied. The small number of palaeoecological studies in Suriname are near the coast; phytolith analysis has not been conducted (Laeyendecker-Roosenburg 1966; Wijmstra 1969). Archaeological remains date the earliest occupation in Surinamese rainforests to around 5,000 calibrated radiocarbon years before present (hereafter cal yr bp) (Versteeg 2003). Pre-Columbian activities (i.e. those occurring prior to European arrival to the Americas in ad 1492) in Surinamese rainforests during the late Holocene likely involved small-scaled slash-and-burn agriculture of cassava (Versteeg 2003). In the late ad 1600s, a sizeable population of enslaved Africans escaped from coastal plantations and eventually settled in various locations in the tropical rainforest interior of Suriname; their descendants are referred to as Maroons (Price 1983; White 2009). Maroon communities today still live a traditional lifestyle that consist of slash-and-burn agriculture and seasonal exploitation of aquatic and land resources in the tropical rainforests (Price 2008; van Andel et al. 2016; van’t Klooster et al. 2018; ESM 1).

Palms are one of the most common and useful plant groups in Amazonia (ter Steege et al. 2013; Muscarella et al. 2020). In the tropical rainforests of Suriname, the palm species *Euterpe oleracea* (locally referred to as Podosiri and in Brazil as Açaí) and eight other palm species are used locally for food, medicine and rituals (van Andel and Ruysschaert 2014). Thus, these extensive and highly diverse forests likely have a complex human history that spans centuries and consists of various forms of palm use. Palms are prolific phytolith producers with morphologies specific to subtribe or often genus (Witteveen et al. 2022), and can thus be used to detect palm enrichment or depletion.

Here, we generate a baseline dataset of phytolith assemblages across four forms of modern land use within the Surinamese rainforests. Using ordination analysis, we assess the variability of phytolith assemblages between the different land uses: (1) cultivation sites; (2) a garden; (3) abandoned fields; (4) tropical forests; and also an archaeological site. We also specifically tested whether: (1) abundances of *Euterpe oleracea* recorded in the modern landscape via unpiloted aerial vehicle (UAV) imagery are positively related to the abundances of phytoliths produced by *E. oleracea*; and (2) aboveground biomass, estimated by MODIS satellite imagery, is positively related to arboreal phytolith percentages. We expect that: (1) phytolith assemblages will be distinguishable between the four forms of land use and the archaeological site; (2) forested sites will contain the highest abundances of arboreal phytoliths, while grass phytoliths will be most abundant at the cultivated sites and garden; (3) abandoned fields and the archaeological site will contain a mixture of grass and arboreal phytoliths; (4) positive correlations between *E. oleracea* crowns and palm phytolith abundances; and (5) aboveground biomass estimates are positively correlated with arboreal phytolith abundances. Our calibration dataset will be a valuable tool in generating models that quantify past land use and the subsequent successional trajectories, so that we can better understand how Amazonian forests respond to anthropogenic pressures on long timescales.

## Material and methods

### Site description and data collection

Suriname lies on the north coast of South America and is in the Guiana Shield. The climate is wet tropical, with a mean annual temperature of 27 °C and mean annual precipitation of 2,200 mm (De Graaf et al. 1999). The region has two wet seasons, the longest one occurring from April to mid-August, and the shorter one occurring in December and January. The dry season runs from September to November and in March (i.e. months containing less than 100 mm precipitation) (Nurmohamed et al. 2007). Biomass estimates in the Surinamese rainforests range from 200 to 490 Mg/ha (Saatchi et al. 2011; Avitabile et al. 2016). Surinamese rainforest soils are mainly composed of ultisols (Jonkers 1987; De Graaf et al. 1999).

**Fig. 1:**
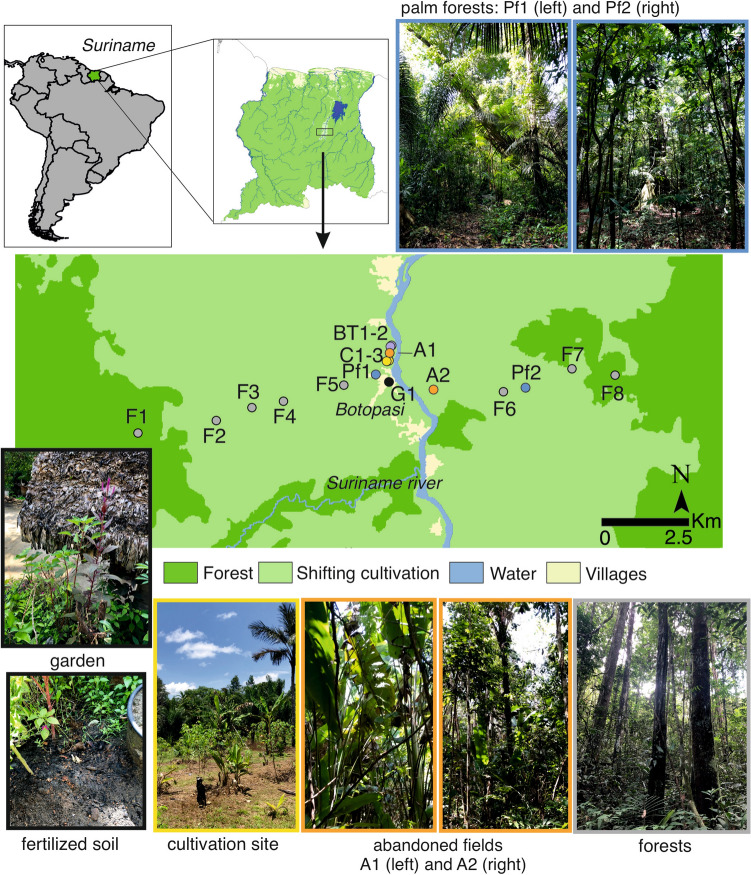
Map showing the sampling sites analyzed in this study, the distribution of shifting cultivation from the National Land Monitoring System of Suriname (2016) and an impression of the local vegetation

Botopasi is a Maroon village that lies in the Sipaliwini district of the Surinamese rainforests along the Suriname River (Fig. 1). Within this region, we collected 17 soil surface samples from a garden (G1), cultivation sites (C1-3), abandoned fields (A1-2), forested sites (F1-8), palm-dominated forested sites (Pf1-2) and an archaeological site (BT1-2) (Fig. 1). The cultivation sites were located on a ca. 0.5 ha plot where crops were being cultivated (ESM 1). Soil surface samples were collected at each of the sites by removing the litter layer and collecting approximately 50 g of material from the top 2 cm of soil (the ‘A’ horizon) within 10 m of the sampling location. At the cultivation sites, a leaf litter sample (C1) was also analyzed (the ‘O’ horizon) to examine differences between phytolith assemblages from the litter on top of the soil (C1) and the top 2 cm of soil (C2) (the ‘A’ horizon) at a single location.

Two of the currently forested sites (BT1-2) were located on an archaeological site called Beng Tapu. Two other forested sites (A1-2) were likely abandoned cultivation sites, indicated by the high abundances of *Attalea maripa* (Maripa), *Heliconia spp.* (Palulu) and *Loxopterygium sagotii* (Slangenhout). These BT and A sites were previously managed, while the other forested F and Pf sites were unmanaged (in living memory), and the garden and cultivation sites were managed. The cultivation site was most intensively managed, as several crops were grown in a specific order (ESM 1), whereas the garden was managed by charcoal fertilization and watering the plants. Two forested sites (F1 and F8) were outside the range of modern shifting cultivation (National Land Monitoring System of Suriname 2016; Sieber et al. 2021; Fig. 1).

Vegetation surveys were not available on the plots, but local guides created a list of the most common taxa present at each site, including their uses (ESM 1 Table 1). We sampled leaves of 26 of these species from Naturalis herbarium (L, U, WAG), The Netherlands, to generate modern reference material for phytolith production from these forests in the upper Suriname River region (ESM 1 Tables 3–5). Four specimens of *Astrocaryum* reported to grow in Surinamese rainforests were sampled that could be the locally known ‘Sapati palm’ (Jonkers 1987; ter Steege et al. 2007). *Oryza glaberrima* (black rice) is used by Saramaccan Maroons for food and ritual purposes and was not currently cultivated, but it is planted during specific months (van Andel 2010; ESM 2).

### Phytolith analysis

For modern reference phytoliths, i.e. those derived from herbarium-collected plant material, we collected 2 cm^2^ of leaf material and burned it at 450 °C for 4 h before chemical processing. For the soil-surface samples collected from the Botopasi region, 1 cm^3^ of unsieved material was processed for phytoliths (the sand fraction was not concentrated and studied).

To prepare phytolith slides, 56,000 microspheres were added to the soil-surface samples. Both the soil-surface and the modern reference samples were soaked on a hot plate with 33% H_2_O_2_ four times, and treated with 10% HCl and KMnO_4_ to remove organic material. Clay material was further removed by decanting the samples. Using bromoform with a specific gravity of 2.3, phytoliths were separated from the remaining soils and mounted on slides using Permount™.

Phytolith slides from modern reference material were counted to 300 phytoliths, if the concentration of phytoliths allowed it. Modern reference samples were counted using a Zeiss Axioscope 5 at 1,000× magnification with immersion oil. The reference samples that contained too few phytoliths, however, were scanned at 630–400× magnification and the presence of morphotypes were noted. Phytolith slides from the cultivation sites, garden and forested sites were counted up to a minimum of 200 arboreal and 400 total phytoliths, at 1,000× magnification using immersion oil. Slides were also scanned for cultivars. Phytolith morphotypes were identified using the latest literature and unpublished guides (Morcote-Ríos et al. 2016; Huisman et al. 2018; Neumann et al. 2019; Witteveen et al. 2022).

### Modern forest characteristics

Mean biomass values were extracted for each sampling site from a raster file containing global estimated biomass values, using the extract function of the package ‘raster’ (Hijmans et al. 2013; Avitabile et al. 2016). Modern palm abundances were quantified using imagery that was captured around our sample sites from a UAV. Our UAV flew at an altitude of 100 m with the camera pointed straight down to capture 1 ha per image. A total of 45 images were captured (5–7 images per sampling site), covering around 37 ha in total. We were unable to capture imagery around sites F3, F4, F5, F6, A, BT, C, and G because of flight prohibitions due to the proximity of an airstrip in Botopasi. Therefore, images were only captured of forested sites > 5 km away from the airstrip (sites F1, F2, F3, F6, F7, F8 and Pf2). One of the palm species, *Euterpe oleracea*, was common in the forests and easily identifiable with the UAV imagery (ESM 2 Fig. 1). Other palms were not as easily identified; therefore, palm crowns were marked either as *E. oleracea* or ‘palm’. Crowns were counted within 20 m, 50 m, 100 m and > 100 m distance from sampling sites, to compare the relationship between palm abundance and palm phytolith abundance at different spatial scales. Four spatial scales were chosen because the spatial signal of phytoliths can vary depending on habitat and edaphic processes (Aleman et al. 2014; Crifò and Strömberg 2020, 2021).

### Data analysis

Phytolith assemblages were plotted and analyzed with morphotypes grouped according to known taxonomy or morphotype (ESM 1 Table 2). Arboreal phytoliths were grouped into categories based on their surface texture (Collura and Neumann 2017; Neumann et al. 2019; Piperno and McMichael 2020). Palm phytoliths were grouped as Conical or Spheroid echinate (SPH_ECH) (Morcote-Ríos et al. 2016). In the Surinamese rainforests, SPH_ECH phytoliths are produced by *Euterpe*, *Oenocarpus*, *Attalea*, *Hyospathe elegans* and *Cocos nucifera* and Conicals are produced by *Astrocaryum*, *Bactris* and *Socratea exorrhiza* palms (Henderson et al. 2019; Witteveen et al. 2022). Several palm morphotypes are indicative of genera and were therefore recorded separately (ESM 1 Table 2). Grass phytoliths were grouped according to subfamily (Piperno 2006; Gallaher et al. 2020). Maize *(Zea mays)* was indicated by Cross 1 phytoliths > 21 μm and Wavy-top rondels (Iriarte 2003; Piperno 2006; Lombardo et al. 2020). Phytoliths and druses were identified from five Zingiberales, including *Heliconia* (Palulu) and Musaceae (banana/bakove), these plants being known to grow in disturbed areas; also phytoliths were found from Marantaceae, which grow in the understory of tropical forests (Chen and Smith 2013).Our overall groupings consisted of five arboreal phytolith types, nine palm types, five types of Zingiberales, and 12 types of grass phytoliths (ESM 1 Table 2).

The 31 phytolith groups were used in a Detrended Correspondence Analysis (DCA) to assess (dis)similarity and distinguishability between the phytolith assemblages. To explore the relationship between palm crowns and palm phytoliths, the Pearson or Spearman correlation was used, depending on the normality of the data. For each spatial scale (20 m, 50 m, 100 m, > 100 m), we assessed the correlations between (1) *Euterpe* palm abundance and SPH_ECH palm abundance and (2) total palm abundance and Total/Conical/SPH_ECH palm phytolith abundance was assessed. To explore the relationship between arboreal phytoliths and biomass, the total abundance of arboreal phytoliths from this study (rugose, rugose ornate, ornate Spheroid and 'Other arboreal' phytoliths) and from literature (McMichael et al. 2012b; Dickau et al. 2013; Heijink et al. 2020; Piperno et al. 2021) were summed (N = 34). The abundance of phytoliths was correlated with biomass values using the Spearman correlation test. All analyses were conducted using the ‘vegan’, ‘raster’, ‘tidypaleo’, and ‘tidyverse’ package in R studio (Dixon 2003; Oksanen et al. 2013; Wickham 2017; Dunnington et al. 2022).

## Results

### Phytolith assemblages from modern reference material

Most phytoliths from the modern reference material have been previously described for arboreal, palm, grass, and Zingiberales species (Piperno 2006; Chen and Smith 2013). A total of eight arboreal morphotypes, ten palms, five Zingiberales, and nine grass phytolith morphotypes were found, along with silica from nondiagnostic plant tissue (Fig. 3).

**Fig. 2:**
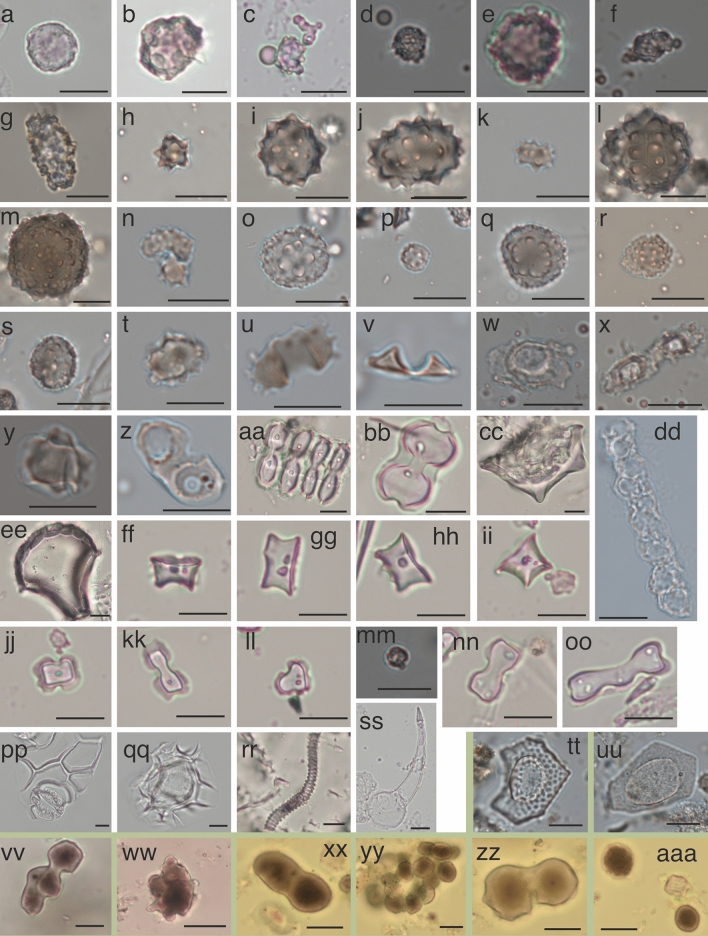
Photographs of phytolith morphotypes from the Herbarium reference material (a-ss), cultivation sites (tt-uu) and garden (vv-aaa). The scale bar is 10 μm. **a** Rugose spheroid, **b** Rugose-ornate spheroid, **c**–**d** SPH_ORN, **e** Nodular spheroid, **f** other arboreal, **g** Ellipsoidal verrucate, **h** SPH_SYM, **i** SPH_ECH, **j** ELL_ECH > 12 μm, **k** ELL_ECH < 12 μm, **l** SPH_ACU > 20 μm, **m** EUT_SPH, **n** Reniform, **o** CON_FEW > 12 μm, **p** CON_FEW < 12 μm, **q** CON_ECH_PRO, **r** CON_ECH, **s** CON_TAB, **t** unknown morphotype of *Attalea sagotii*, **u**–**v** T1 trough, **w**–**x** T2 trough, **y** druse, **z** unknown type A, **aa**–**bb** scooped Bilobate, **cc** peaked glume, **dd** unknown silica, **ee** BUL_FLA, **ff**–**ii** Rondel, **jj** Cross 2, **kk** Cross 7, **ll** other Cross, **mm** Psilate spheroid, **nn** Bilobate, **oo** asymmetrical Polylobate, **pp** epidermis, **qq** hair base, **rr** Tracheary, **ss** ACU_BUL, **tt** *Cyperus/Kyllinga*, **uu** *Commelinaceae,* **vv**–**yy** unknown burnt clumps, **zz** burnt Spheroid palms, **aaa** burnt Psilate spheroid

Four of the 11 tree species recognized by local guides produced arboreal spheroid phytoliths (ESM 1 Table 3). *Couepia guianensis* (Apesie) contained the most phytoliths, mainly Ornate and Rugose spheroids < 10 μm, and 1 small nodular spheroid (Fig. 2a–f). *Swartzia longicarpa* (Bugu bugu) contained small & large Rugose spheroids and 1 large Granulate spheroid. *Dicorynia guianensis* (Basralocus), a species widely used for house construction and to make Maroon pottery in the colonial and contemporary period (Stahel 1944), contained small Rugose spheroids, 1 large Granulate spheroid, and 1 Ellipsoidal verrucate (Collura and Neumann 2017; Fig. 2g). S*pondias mombin* (Mope) contained Rugose and Ornate spheroids. *Goupia glabra* (Kopi), *Inga alba* (Abonkini), *Eperua falcata* (Walaba) and *Cecropia* species produced only a few non-diagnostic phytoliths. *Loxopterygium sagoti* (Slangenhout) and *Ceiba pentandra* (Kankan tree), a tree with a spiritual value and associated to locations indicative of sedentism, did not produce phytoliths (ESM 1 Table 3).

All reference material from the identified palms and Zingiberales that grow at the sampling sites produced phytoliths (ESM 1 Tables 3–5). *Astrocaryum* specimens produced Conical morphotypes with few or many projections between 8 and 17 μm (Fig. 2o–s, Tables S4-S5), which were previously described for *Bactris* and *Socratea exorrhiza* (Witteveen et al. 2022). Spheroid morphotypes were produced by *Attalea maripa, Attalea sagotii, Cocos nucifera* and *Euterpe oleracea* (Morcote-Ríos et al. 2016; Witteveen et al. 2022, Fig. 2h–k). Outside of the count, *Euterpe oleracea* produced SPH_ACU > 20 μm and EUT_SPH (Fig. 2l–m), like *Euterpe precatoria* (Huisman et al. 2018; Witteveen et al. 2022). *Attalea sagottii* also contained CON_BAS and a mixture between ELL_ECH and CON_BAS (Fig. 2t). *Heliconia* contained T1 trough phytoliths and Musaceae species T2 trough and druses (Chen and Smith 2013, Fig. 2u–y). Strings of silica and unknown type A phytoliths were also found in *Heliconia* and Musaceae (ESM 1 Table 2; Fig. 2z, dd).

The reference material of identified Poaceae species was abundant in phytoliths. *Oryza glaberrima and Oryza sativa* produced scooped Crosses (11–34%), scooped Bilobates (54–80%), BUL_FLA and epidermis (Fig. 2aa–bb, ee). Peaked glumes were only found in *Oryza sativa* (Fig. 2xx). *Cymbopogon citratus* produced Rondels, Polylobates, and Crosses (Fig. 2ff-ll, nn-oo). Most Crosses of *C. citratus* were small and irregular, with only three lobes instead of four (Fig. 2ll).

### Phytolith assemblages from soil-surface samples

**Fig. 3:**
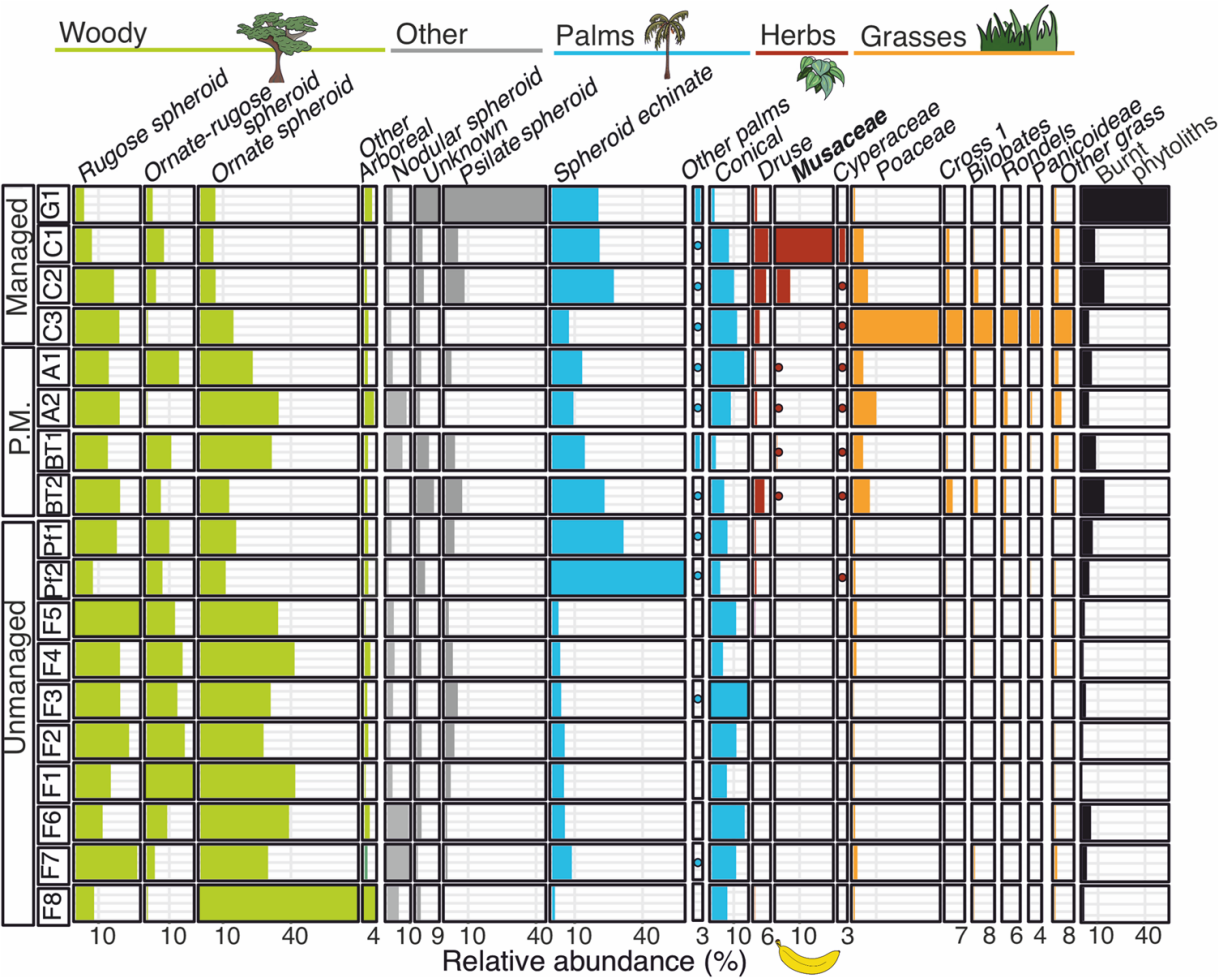
Phytolith assemblages from the garden (G1), cultivation sites (C1-3), abandoned fields (A1-2), archaeological site (BT1-2) and forested sites (F1-8, Pf1-2) in relative abundance (%). Colours and icons indicate different plant groups, P.M. stands for previously managed, cultivars are in bold

**Fig. 4:**
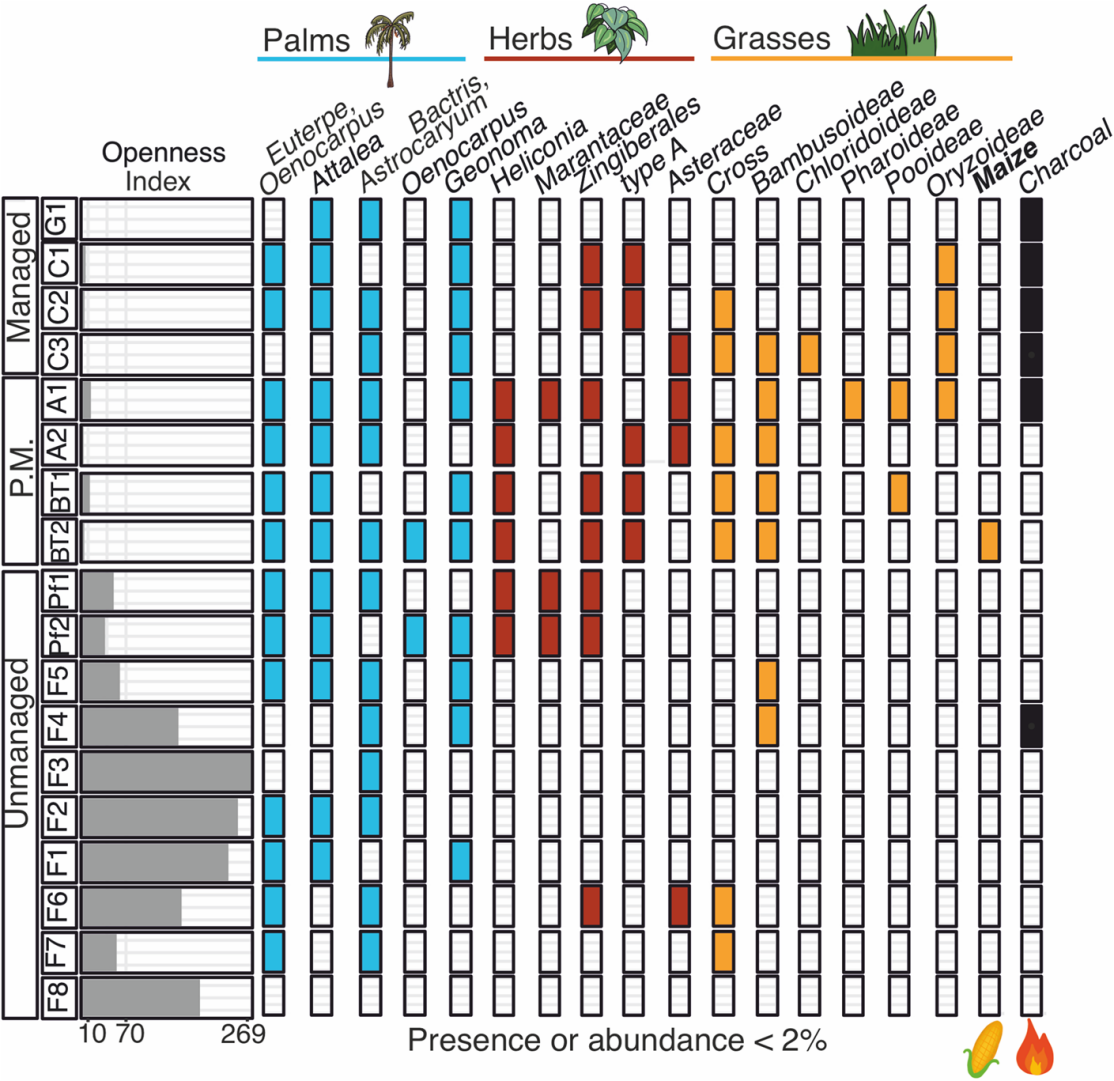
Phytolith assemblages from the garden (G1), cultivation sites (C1-3), abandoned fields (A1-2), archaeological site (BT1-2) and forested sites (F1-8, Pf1-2) in presence or abundance < 2%. Colours indicate different plant groups, P.M. stands for previously managed, cultivars are in bold**.** The openness index is also shown

The garden and cultivation samples were collected in areas that were being actively managed, and their phytolith assemblages were clearly distinguishable from unmanaged sites (Figs. 3, 4). The differences between managed and unmanaged sites resulted mainly from higher abundances of Psilate spheroid and burnt phytoliths in the garden and higher abundances of Zingiberales and Poaceae phytoliths at the cultivation sites (whereas unmanaged sites contained high abundances of arboreal spheroids) (Figs. 3, 4).

Phytolith assemblages of cultivation sites (C1-3) were mainly composed of grasses (4.4–37.3%), palms (19.5–22%), and Zingiberales (2.1–31.6%) (Fig. 3). Grass phytoliths from Panicoideae, Chloridoideae*,* Pooideae*,* Pharoideae and Oryzoideae were likely from weeds, crops (*Saccharum officinarum, Oryza sativa* or *glaberrima*), and useful species (*Pharus sp.*) (Maezumi et al. 2018; ESM 1 Table 1). Musaceae cultivars were present in all samples (where they were grown), but in C3 these phytoliths were only encountered during an extended scan, and not during the counting of the phytolith sum (of 400 total phytoliths). No extra large (> 21 μm) Cross 1 or Wavy-top rondel phytoliths indicative of *Zea mays* (maize) were found, and maize was not being cultivated (ESM 1 Table 1). Druses (> 2%) were probably from rhizomes (Yost 2018). Phytoliths from *Cyperus/Kyllinga* (Cyperaceae) and Asteraceae (C3) were present (< 2.5%); these plants are often abundant in disturbed or open areas (Piperno 2006; Dickau et al. 2013; Testé et al. 2020; Watling et al. 2020). The tropical herb Commelinaceae and unknown Type A from the Zingiberales reference material were also found during an extended scan, outside the phytolith sum (Fig. 2z, uu). Palm phytoliths came from the understory palm *Geonoma* and from the canopy palms *Attalea maripa*, *Euterpe*, *Oenocarpus* and *Bactris*, which are used for food and construction (Fig. 4; ESM 1 Table 6). Local guides only identified *Attalea maripa*, *Astrocaryum* and *Bactris* in the cultivation sites (ESM 1 Table 1). Between 4 and 14% of the phytoliths were burned and the openness index ranged between 0 and 6.25 (Fig. 4).

*Cymbopogon citratus* was the only plant in the garden that produced phytoliths (ESM 1 Table 1, Fig. 2ff–oo), but its morphotypes were not found (Figs. 3, 4). Instead, the assemblage was dominated by Psilate spheroid (Fig. 2mm), produced by many woody taxa and monocots, unknown morphotypes, and SPH_ECH palms produced by *Euterpe, Attalea, Oenocarpus, Cocos nucifera,* and *Hyospathe elegans*. The unknown phytoliths were clumped or burned (Fig. 2vv–aaa). Arboreal phytoliths occurred in low percentages of < 17%. Phytoliths of Musaceae, Commelinaceae, Cyperaceae and grass phytoliths of Chloridoideae*,* Pooideae*,* Pharoideae and Oryzoideae were not found, only “other grass” phytoliths were observed (< 1%). Druses were probably from rhizomes (Yost 2018). Asteraceae occurred outside the phytolith sum. Conical palms from *Bactris*, *Astryocaryum* and *Geonoma* were almost absent (< 1.5%). Many phytoliths (54%) were burned. The openness index could not be calculated, because grass silica short cell phytoliths (‘GSSCPs’) were absent.

The phytolith assemblages of the abandoned fields (A1 and A2) contained grass percentages (4–10%) similar to cultivation sites (C1-3) and archaeological (BT) sites (Fig. 3), Conical phytolith abundances were similar to forest (F) sites (< 15%) and SPH_ECH palm phytolith abundances were slightly higher than F sites (10–13%). Morphotypes of *Bactris, Attalea maripa,* and *Astrocaryum* correspond with identifications by local guides (ESM 1 Table 1). Also *Euterpe, Oenocarpus* and *Geonoma* (A1) phytoliths were found in low abundances (< 1%). Pharoideae and Oryzoideae grass phytoliths (A1) were likely of useful species and cultivars (Maezumi et al. 2018), but maize phytoliths were not found (Fig. 4). High percentages of nodular phytoliths (8.5% at A2) were produced by Zingiberales and Malvaceae (Watling and Iriarte 2013; Piperno and McMichael 2020). Druses from Zingiberales were present, but *Heliconia* and Musaceae were only found during an extended scan outside of the phytolith sum. Cyperaceae phytoliths were present at < 1% and Asteraceae phytoliths were found outside of the phytolith sum at A2. Burned phytoliths occurred between 4 and 6% and the openness index ranged between 3 and 15 (Figs. 3–4).

The phytolith assemblages from an archaeological site Beng Tapu (BT1 and BT2), contained grass percentages similar to the C and A sites (4–7%) (Fig. 3). At BT1, those grasses came mainly from other grasses and at BT2 from Cross 1 and Bilobates. Phytoliths of Bambusoideae, Pooideae (BT1), and maize (BT2) were identified during an extended scan (Fig. 4). High percentages of palms (19–32%) included *Attalea, Euterpe, Oenocarpus, Geonoma, Bactris* (BT2) and *Astrocaryum* (BT2). Unknown phytoliths were higher than at other sites, except in the garden, and included fused clumps (Fig. 2vv-xx). *Heliconia,* Musaceae, Zingiberales and Unknown type A phytoliths were found. BT2 was abundant in Zingiberales (4.8%), originating from druse phytoliths. Cyperaceae phytoliths were present at < 1% but Asteraceae*,* Commelinaceae, Chloridoideae*,* Pharoideae and Oryzoideae were not found. Burned phytoliths occurred between 9–14% and the openness index ranged between 4.2 and 13 (Figs. 3, 4).

Forest (F) sites contain higher abundances of arboreal phytoliths (> 71%) and lower abundances of grass and herb phytoliths (< 2%) and spheroid palms (< 10%) than C, G, A and BT sites. Rugose spheroid phytoliths are produced most abundantly by Chrysobalanaceae and by families Lecythidaceae, Moraceae, Malvaceae and Proteaceae (Piperno and McMichael 2020). Ornate spheroid phytoliths are produced by Acanthaceae, Burseraceae, Lecythidaceae, Malvaveae, Moraceae, Violaceae, and Vochysiaceae (Piperno and McMichael 2020) and occur in Suriname (Funk et al. 2007). The phytolith assemblage from F8 was almost completely composed of Ornate spheroid (68.8%). Chrysobalanaceae likely produced the high abundance of arboreal phytoliths at F7 (ESM 1 Table 1). Forest understory Zingiberales correspond with ‘other Zingiberales’ phytoliths at F3, F4 and Pf1, druses at F5, F8, Pf1 and Pf2. The low percentages of grasses came from Bilobate at F4, F5, F7 and F8, from Rondel at F1-3 and Pf1, from Bambusoideae at F4-5 and from other grass phytoliths at all forested sites. No phytoliths of Musaceae, Asteraceae*,* Commelinaceae, Cyperaceae and grasses Chloridoideae*,* Pooideae*,* Pharoideae*,* and Oryzoideae were found. Few phytoliths (< 7%) were burned and the openness index of the forested sites was between 37 and 269.

Forests with high palm abundances (Pf1 and Pf2) differed from the F sites due to the high percentages of palms (> 30%), low abundances of Rugose (7.5–18%) and Ornate spheroid (< 16%) and the presence of *Heliconia* (< 1%). *Euterpe oleracea* was present at Pf2, and although *Euterpe* specific phytoliths (ESM 1 Table 2) were only observed during an extended scan, the symmetrical spheroids that this species produces in abundance dominated the assemblage (Morcote-Ríos et al. 2016; Witteveen et al. 2022). Conical palms produced by *Bactris, Socratea exorrhiza* or *Astrocaryum* composed between 4.3–13.7%, similar to the C and A sites. *Geonoma* was not identified by local guides but its phytoliths occurred at F1, F4, F5 and Pf2. Phytoliths of *Attalea maripa* were seen in F8, F1, F2, F5 and Pf1, but only identified in Pf1. The phytoliths of *Euterpe* and *Oenocarpus* were present in F1, F2, F5, Pf1 and Pf2, but *Euterpe* was only identified in Pf1 and *Oenocarpus* was not identified by local guides (ESM 1 Table 1).

**Fig. 5:**
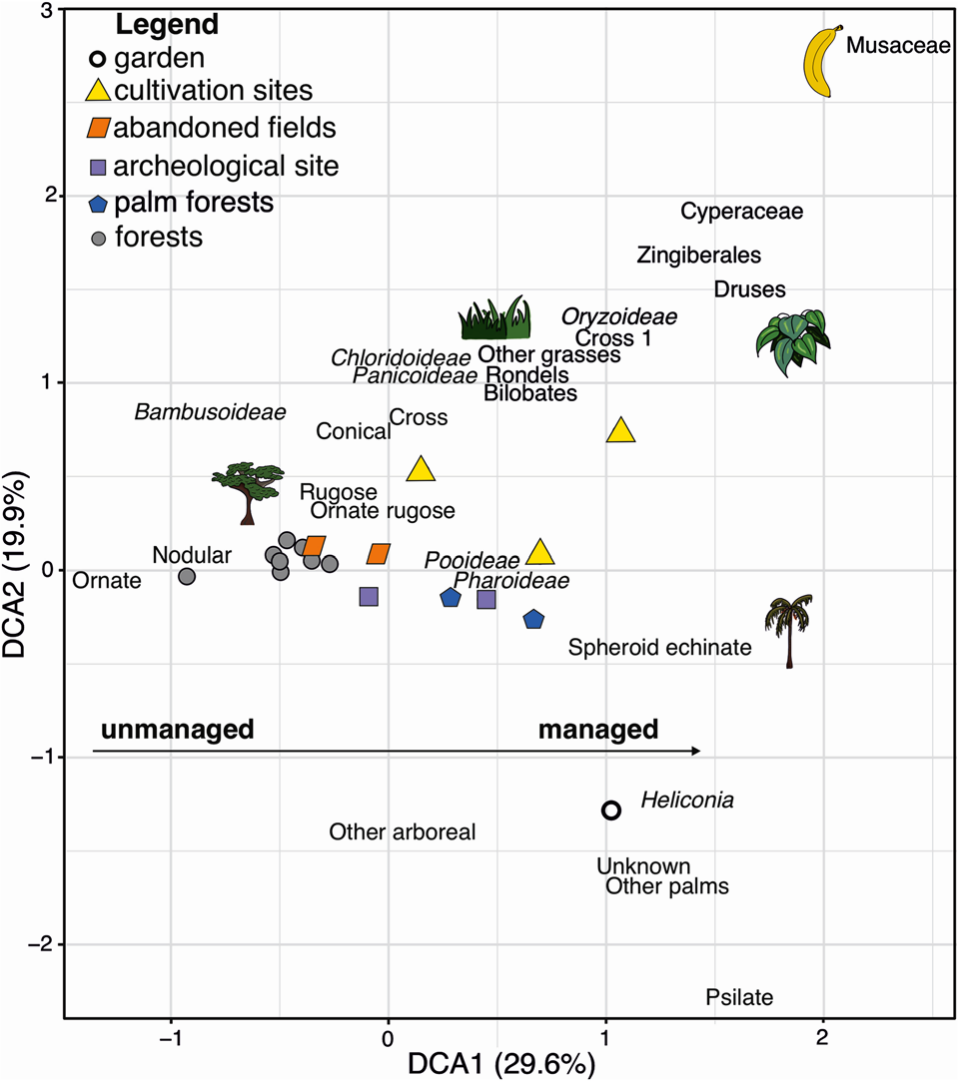
Detrended Correspondence Analysis (DCA) of phytolith percentage data from all the sampled sites, with DCA1 explaining 29.6% of the variance and DCA2 explaining 19.9%. Sites are colour and symbol coded (see legend), and icons represent the types of plants associated with the phytolith morphotypes

The DCA results show the largest difference in phytolith assemblages between currently managed versus unmanaged sites (DCA1; Eigenvalue = 0.296; Fig. 5). The second DCA axis (DCA2; Eigenvalue = 0.199) separates the cultivation sites from the garden, with the forested sites in between. Associated with forested sites are Bambusoideae grasses and all arboreal phytoliths except ‘Other arboreal’, which is near the garden with, ‘Other palms’, ‘unknown’, psilate and *Heliconia* phytoliths. Phytoliths associated with cultivation sites are CONICAL palms, Cyperaceae, Druses, Zingiberales, Musaceae and all grasses except Bambusoideae. The palm forests and archaeological site (BT2) are on the negative axis of DCA1 with the managed sites, probably due to the high abundances of SPH_ECH palms, and the presence of Pooideae and Pharoideae grasses in BT2. Litter sample C1 stood out from the rest of the assemblages due to high Musaceae phytoliths (24%) and the garden due to high psilate abundances (43%) (ESM 2 Fig. 2).

### Correlations of phytolith assemblages with modern forest characteristics

**Fig. 6:**
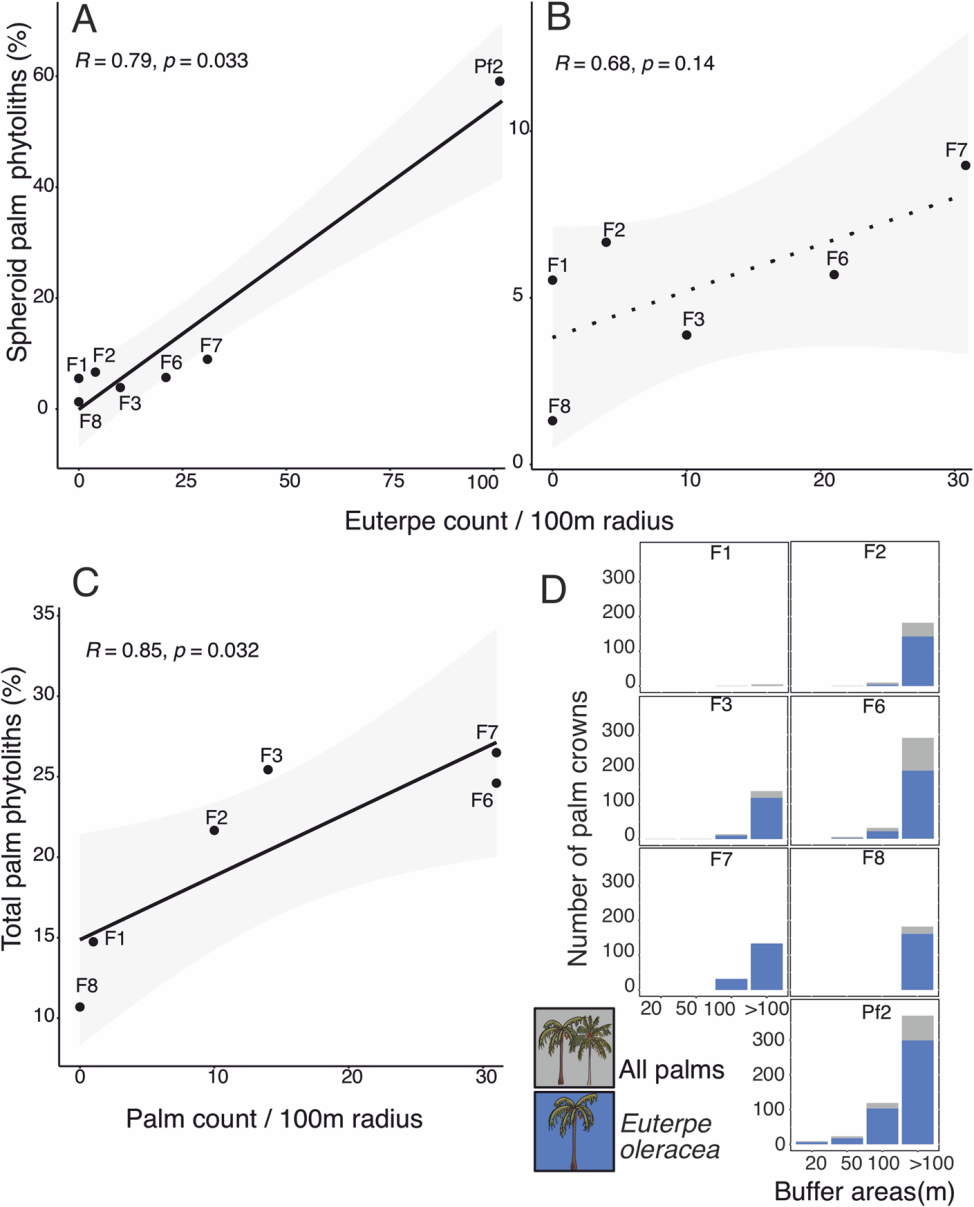
**A**, **B** Correlation of the number of *E. oleracea* crowns and Spheroid echinate palm phytoliths (%) within 100 m buffer. **A** with site Pf2 included, and **B** Pf2 excluded, **C** correlation of the number of total palm and total palm phytoliths (%) within 100 m buffer, shown without site Pf2, and **D** the distribution of palm crowns within 20, 50, 100 and > 100 m buffer sizes for total number of *E. oleracea* and other palm crowns

We recorded ranges of *E. oleracea* abundances from 0 to 6, 0 to 17, 0 to 102 and 0 to 296 at the 20 m, 50 m, 100 m and > 100 m buffer sizes (Fig. 6D). Total palm crowns (including *E. oleracea)* were 0 to 9, 0 to 23, 0 to 199 and 0 to 370 at the 20 m, 50 m, 100 m and > 100 m buffer sizes (ESM 1 Table 7). *E. oleracea* produces SPH_ECH phytoliths (ESM 1 Table 2), so correlations were only made between these types and the abundances of *E. oleracea* in the landscape (Fig. 6A–B). Because palm crowns were not evenly distributed between sites (only Pf2 was abundant in palms), correlations were also performed without site Pf2 (ESM 1 Table 7).

Pearson or Spearman correlation tests indicated that SPH_ECH phytolith percentages were positively and significantly correlated with the number of *E. oleracea* crowns for the buffer size of 100 m, but not for 20 m, 50 m and > 100 m (ESM 1 Table 7). An increase from 31 to 102 *E. oleracea* crowns corresponded with an increase in 50% SPH_ECH phytoliths. Without site Pf2, there was no significant correlation between the SPH_ECH phytoliths and the *Euterpe* crowns. But the total palm phytoliths and the total palm crowns were positively and significantly correlated for the buffer size of 100 m without Pf2 (Fig. 6C). Increases from 0 to 31 total palm crowns corresponded with a 10–15% increase in total palm phytoliths.

**Fig. 7:**
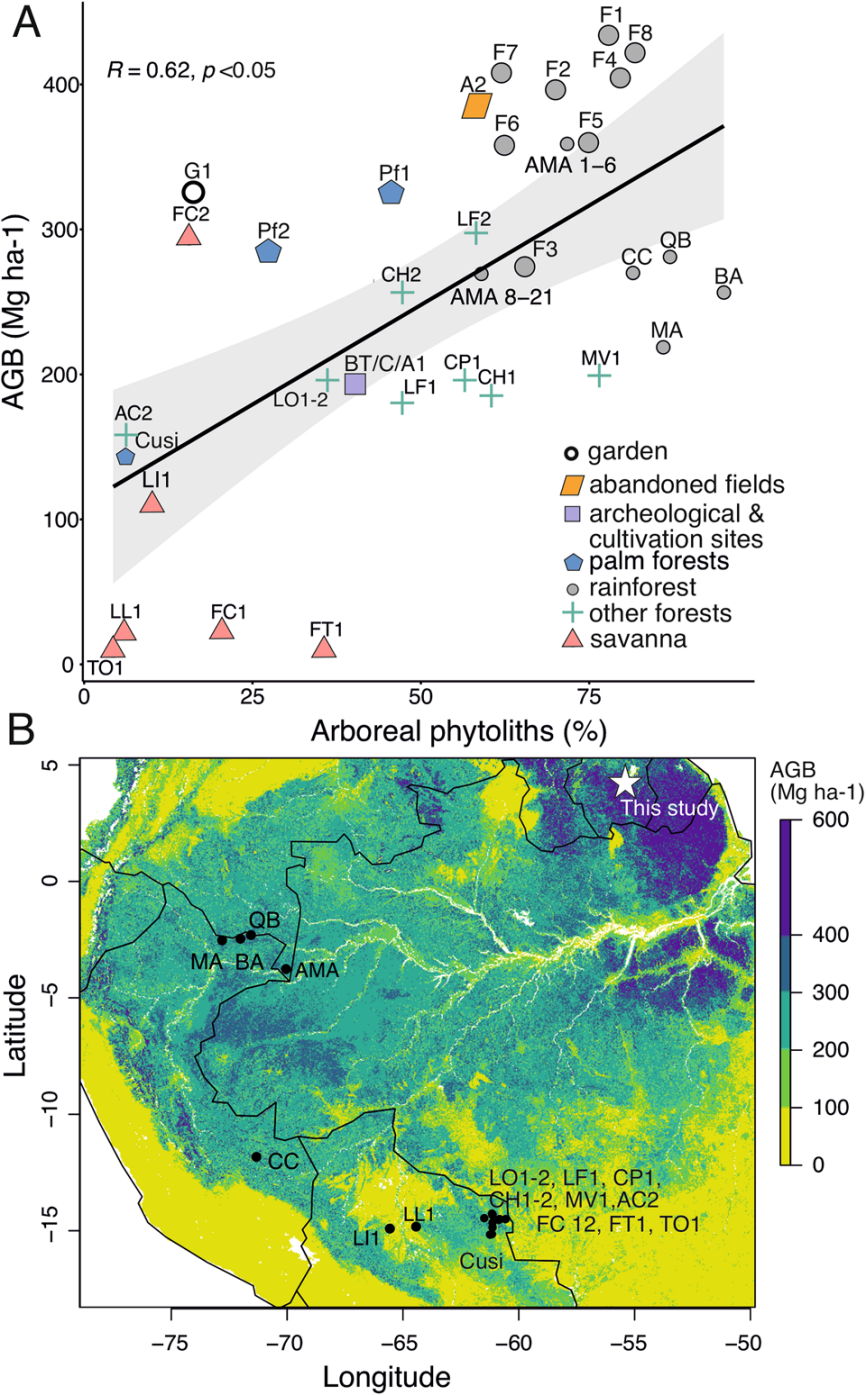
A The positive correlation of arboreal phytoliths in percentages and aboveground biomass (AGB) in Mg ha^−1^ (Avitabile et al. 2016). Different symbols and colours indicate the vegetation or land use of the sampling sites, which are from this study and McMichael et al. 2012b; Dickau et al. 2013; Heijink et al. 2020; Piperno et al. 2021. **B** Map of sampling sites plotted on aboveground biomass (AGB) in Mg ha^−1^. Our sampling sites are indicated by a star symbol, and published sites are shown as circles

The abundance of arboreal phytoliths across Amazonia was significantly and positively correlated with biomass estimates (R = 0.62, p = 8.2e-05) (McMichael et al. 2012b; Dickau et al. 2013; Avitabile et al. 2016; Heijink et al. 2020; Piperno et al. 2021; Fig. 7; ESM 1 Table 8). Most forested sites have high biomass values > 275 Mg ha^−1^ and > 50% arboreal phytoliths, whereas most savanna sites have low biomass < 110 Mg ha^−1^ and < 36% arboreal phytoliths. The other forested sites (evergreen, semi-deciduous, or liana) reflect intermediate values of biomass (between 180 and 200 Mg ha^−1^) and intermediate percentages of arboreal phytoliths (36–76%). Sites P1, 2, 3, BT1, BT2, and A1 have the same biomass value due to their close proximity and 1 km resolution of the biomass data. Unlike the palm-dominated sites in this study, the TF palm forest in Bolivia has low biomass values and low arboreal phytoliths. In general, disturbed forests are separated from closed canopy forests and savanna sites. The garden and FC2 have high estimated biomass values (> 293 Mg ha^−1^), and low arboreal phytoliths (< 17%) because the scale of the site (and opening) is much smaller than the 1 km pixel value for biomass.

## Discussion

Phytolith assemblages are known to vary among major forest types in Neotropical humid and dry forests and savannas (Dickau et al. 2013; Testé et al. 2020; Watling et al. 2020; Crifò and Strömberg 2021). Our data and analyses show that phytolith assemblages also vary among land use types on local scales within tropical rainforests, and that managed landscapes are clearly distinguishable from unmanaged ones (Figs. 3, 4, 5). That phytolith assemblages can reflect such a gradient of modern land use demonstrates their potential in palaeoecological reconstructions of successional trajectories after site usage and abandonment. Using the principle of uniformitarianism (the present is the key to the past), phytolith assemblages from past soil samples similar to those from managed sites are likely indicative of past human activities (Lyell 1854).

### Phytolith assemblages from modern reference material

Phytoliths of modern reference material were almost all found in soil surface samples, showing that the phytolith assemblage corresponds well to the local vegetation of phytolith-producing taxa (Dickau et al. 2013; Watling et al. 2020). Most of the tree species in the Surinamese rainforests, however, produced few or no phytoliths. Therefore, differences in the abundance of the most common tree species over time would not be recorded in the phytolith record. Also, increased abundances of useful tree species over time could be missed because similar phytoliths are produced among woody taxa. The arboreal phytoliths from the forested sites are likely from a few taxa of trees and shrubs that produce phytoliths (Piperno 2006; Piperno and McMichael 2020). The early successional taxon *Cecropia* does not produce phytoliths, but the increase in grasses and Zingiberales following canopy openings would be detected. For example, unknown type A phytoliths produced by Musaceae and *Heliconia* were counted at the managed and previously managed sites only.

The palms *Astrocaryum* and *Bactris* produce similar phytoliths and therefore these palms cannot be distinguished in the phytolith record. *Socratea exorrhiza* and *Astrocaryum* also produce a similar morphotype (CON_FEW) (Witteveen et al. 2022). The odd Conical phytoliths produced by *Attalea sagotii* could be a contamination, because Spheroid and Conical morphotypes are usually not produced by the same species. Due to the overlap of palm morphotypes produced between taxa, this study grouped SPH_ECH and Conical morphotypes and used the presence of specific morphotypes as an additional indicator of the “presence” of a specific genus. Peaked glumes were not found in reference material for *Oryza glaberrima*, likely because this morphotype is rare (Radomski and Neumann 2011). Most of the morphotypes produced by *C. citratus* were typical for Panicoideae grasses. The three lobed Cross, however, may be specific to the genus, as most crosses have four lobes (Piperno 2006).

### Phytolith assemblages across a gradient of land use

Corresponding with our hypothesis, forested sites contain higher amounts of arboreal phytoliths than managed sites. This and other Neotropical studies show that high abundances of arboreal phytoliths (> 60%) and low abundances of phytoliths from grasses and herbs (< 4%) reflect closed canopy forested settings (Dickau et al. 2013; Heijink et al. 2020; Watling et al. 2020; Piperno et al. 2021). The cultivation sites, an open field of 0.5 ha, and the previously managed sites, covered with successional vegetation, contained > 4% of grass and herb phytoliths (Fig. 3). What has been reconstructed as forested in Amazonian soil studies from other regions analyzed also have rare Poaceae (except Bambusoideae), as do associated modern surface phytolith assemblages from censused vegetation (McMichael et al. 2012b; Piperno et al. 2021). Our results also confirm previous studies from high biomass regions of the northwestern Amazon (Fig. 6B) where soil core samples contained a lack of grasses, herbs, and cultivars, and were interpreted as having little to no evidence of past forest opening (McMichael et al. 2012a, b, 2015; Heijink et al. 2020; Piperno et al. 2021).

Palm-dominated forested sites were not separated from managed sites in the DCA but were differentiated from the other forested sites (Fig. 5). Previous studies have shown that *Euterpe* can dominate the phytolith assemblage in flooded forests (Dickau et al. 2013), but ordination did not separate palm forests (mostly *Attalea* sp.) from other forests in Brazil (Watling et al. 2020). Across the Neotropics, palms can comprise up to 60% of the phytolith assemblage of closed canopy forests (Dickau et al. 2013; Heijink et al. 2020; Crifò and Strömberg 2021). Overall, phytoliths seem to provide a sensitive proxy for local palm abundances, but high abundances of (useful) palm phytoliths alone are not sufficient to indicate land use, as even useful species of palms are naturally abundant in Neotropical forests. The lack of vegetation surveys might explain the presence of palm phytoliths that were not identified by local guides, as *Geonoma* grows in Suriname (ter Steege et al. 2007), but was not listed as a useful species nor identified. These phytoliths could also be false positives, as palm phytoliths have been reported to occur in soils without local palms present (Watling et al. 2020), and surface samples can often retain the last several hundred years of history (Piperno 2006).

Unlike the DCA, the openness index (Fig. 4) detected the difference between unmanaged forested sites (F and Pf), previously managed (A and BT) and currently managed land (C and G), likely because palm phytoliths were excluded from this index. The openness index has also detected deforestation after European colonization in the Galapagos (Astudillo 2018). The disturbed, open forested vegetation at previously managed sites (A and BT) were reflected by the high abundances of grass, herb and Zingiberales phytoliths (> 5%). The presence of cultivars (Musaceae, maize) and weedy taxa reflected past land use. Although these systems are not directly comparable, a similar mixture of arboreal and grass phytoliths were found on abandoned fields in the Galapagos (Astudillo 2018). Despite shared past disturbance, DCA detected the differences in modern vegetation of A1-2 and BT1-2 as more forested sites A2 and BT1 are closer to other forested sites (ESM 1 Table 1, Fig. 5). Although A1 was dominated by *Heliconia* spp. (Palulu), T1 trough morphotypes were rare and the assemblage instead reflected past land use (Fig. 4). Combining DCA and the openness index is useful to differentiate successional trajectories in tropical forests after (past) land use.

Contrary to our expectations, the garden sample contained few grasses. The phytolith assemblage may be derived from soils used for fertilization (Fig. 1), explaining the high abundance of burnt phytoliths, palms, and unknown clumps of phytoliths found in the garden sample, along with charcoal (Figs. 2, 4). Similar burnt clumps of palms and unknown phytoliths occur at BT2 and have been found in archaeological sites in Amazonia and the Caribbean (Watling et al. 2015; Pagán-Jiménez et al. 2020; Elliott et al. 2023). In those settings, such phytoliths were interpreted as remnants of past burning activities and palm use. Another explanation for the lack of grass phytoliths in the garden can be weed removal. In the managed sites, there was a relatively high abundance of burned phytoliths and druses, which can be useful to distinguish land use types (Fig. 3).

Our results show that many locally grown cultivars are well detected using phytolith analysis. Phytoliths of cultivars were found only in the cultivation sites and previously managed A and BT sites (Figs. 3, 4), where they are (or were) grown locally. The phytoliths of these cultivars were not found at sites where they were not being grown. Samples from managed soils also contained Asteraceae*,* Cyperaceae*,* Commelinaceae*,* Chloridoideae*,* Pooideae and Pharoideae phytoliths. This combination of phytolith types was not found in unmanaged sites and indicates farming in Surinamese rainforests. Such key phytolith taxa, however, should be interpreted in the context of the local setting, as they can also indicate wetlands or savanna vegetation (Iriarte et al. 2010; Dickau et al. 2013). But in closed canopy rainforests, these taxa likely indicate (human) disturbance. This unique combination of phytoliths likely also represents farming practices in other areas of Amazonian rainforests, though this hypothesis needs further testing.

Musaceae phytoliths have been found in palaeoecological reconstructions in other Neotropical settings (e.g. Ecuador and the Dominican Republic) (Astudillo 2018; Castilla-Beltrán et al. 2020). Musaceae were also found at the archaeological site (BT), in an area where Maroons lived during the colonial period. Musaceae are an Old-World cultivar and are not natively found in the Neotropics (Daniells et al. 2001; Häkkinen and Sharrock 2002; Carney and Rosomoff 2011). Therefore, Musaceae phytoliths can be used as a dating control point to indicate colonial periods (and farming activities) (Castilla-Beltrán et al. 2018). Our results confirm that the detection of Musaceae phytoliths is very high in areas where it is currently being grown, confirming that the presence of these phytoliths can be a powerful chronological control for colonial periods.

### How phytoliths reflect the modern environment

The high abundance of *E. oleracea* crowns at Pf2 and low abundances at F sites was accurately reflected by the abundance of SPH_ECH phytoliths within 100 m. This significant correlation was probably driven by the high palm crown abundance in Pf2, because total palm count and total spheroid phytoliths within 100 m reflect the same R and p-values (ESM 1 Table 7). Local guides identified *E. oleracea* only near Pf2 and F6 (ESM 1 Table 1). Other palms than *E. oleracea* probably contributed to the phytolith assemblages at the F sites, because without Pf2 included, the correlation between *E. oleracea* and SPH_ECH phytoliths was no longer significant. Instead, there was a significant positive relationship with total palm count and total palm phytoliths (within 100 m) and total palm count and conical palm phytoliths (within 50 m). The understory palms are not visible on UAV images but likely contributed to the phytolith assemblages as the forests without canopy palms consisted of up to 15% total palm phytoliths (Fig. 6). Most sites did not contain canopy palms within 20 and 50 m buffer areas (Fig. 6D), and only Pf2 had high palm abundances, making it difficult to predict patterns. Future research should include more sites with intermediate and high palm abundances to improve quantifying palm abundances using phytoliths, as the relationship may not be linear. Also, vegetation surveys or information on the abundance of understory palms should be considered.

Arboreal phytoliths were able to detect differences in estimated biomass between forests and savannas (Fig. 6). It is likely that phytoliths represent local changes in biomass, because phytoliths can also be used to quantify canopy cover in subtropical forests and tree cover density in a savanna-forest transition (Bremond et al. 2008; Li et al. 2022). The correlation between arboreal phytoliths and biomass is likely smoothed out and lacks detailed patterns, because biomass values were extracted from a 1 km-resolution dataset. The forested sites in Suriname have lower arboreal phytoliths than the forest plots of Peru (McMichael et al. 2012b; Piperno et al. 2021), but higher estimated biomass values. This discrepancy between arboreal phytoliths and biomass estimates may be explained by (1) the coarse-resolution dataset, (2) arboreal phytoliths from the sand fraction were not included here, which could have been higher in Peru and (3) the abundance of palms, as palms were not included in the biomass estimates but are common in western Amazonia (Muscarella et al. 2020). Estimating the biomass of palm-dominated forests (like Pf1-2) using only the abundance of arboreal phytoliths may be insufficient, as palm phytoliths can dominate the assemblage. These results highlight the need for accurate, high-resolution environmental and vegetation data for calibration studies. Future research can refine these correlation estimates based on improved biomass data, but these first results show that arboreal phytoliths can be used to quantify biomass changes across different vegetation types.

### Quantifying past environmental changes and recovery

This study has shown the potential of phytoliths to quantify local palm abundances and biomass changes (using UAV and satellite observations). In Suriname and many other areas of Amazonia, (long-term) vegetation monitoring is lacking because ground-based surveys are expensive, time-consuming, and labor intensive. Unpiloted aerial vehicles can capture high-resolution data on vegetation composition and structure, making them an attractive tool for aerial surveys. Especially for a local proxy like phytoliths, high-resolution data are important for accurate calibrations. Therefore, UAVs have a high potential to correlate microfossil assemblages with modern environmental conditions. But optimal flying speed and altitude should be explored to capture images that are most useful for gathering environmental and vegetation data while covering the largest possible area.

We highlight the potential of using our calibration dataset as a foundation for quantifying past human activities and long-term forest recovery in Amazonia. If future palaeoecological and archaeological studies in the region can quantify past changes in palm abundances and biomass, global carbon models can be improved. As Amazonia is an important carbon sink, improved models are crucial to mitigate climate change (Brienen et al. 2015; Hubau et al. 2020). Following temperate studies, the relative role of human activities and climate change as drivers of past vegetation could be explored. Additionally, quantifying past vegetation changes would help to gain insight into the ecological legacies of past human activities in Amazonia (Levis et al. 2017; Piperno et al. 2019; McMichael 2021).

## Conclusions

This study aimed to improve the detectability of past human activities in tropical forests using phytolith analysis. Our results show that phytolith assemblages vary among a gradient of human disturbance (forested sites, abandoned fields, an archaeological site, garden, and cultivation sites) on local scales and can be used in palaeoecological reconstructions of successional trajectories after site usage and abandonment. Our results separated managed landscapes from unmanaged ones, and the openness index further differentiated unmanaged from forested sites that were previously managed. A combination of phytoliths from Musaceae, *Oryza*, Asteraceae, Cyperaceae, Commelinaceae, Chloridoideae, Pooideae and Pharoideae probably indicated farming activities. Finally, this study also provided the building blocks needed to begin quantifying past vegetation using phytoliths, shown by significant correlations between components of the phytolith assemblages with the modern abundances of *Euterpe* (Podosiri) obtained from UAV imagery and with estimates of aboveground biomass obtained from satellite imagery.

### Supplementary Information

Below is the link to the electronic supplementary material.

Supplementary file1 (XLSX 44 KB)

Supplementary file2 (DOCX 1297 KB)

## Data Availability

The phytolith data is available as supplementary material.
