# Supplementary material for: Phytolith assemblages reflect variability in human land use and the modern environment
Source: Veg Hist Archaeobot. 2023 Jun 27;33(2):221–36. doi: 10.1007/s00334-023-00932-2 (PMC10884070; doi:10.1007/s00334-023-00932-2)
Supplement: Supplementary file 2 — Supplementary file2 (DOCX 1297 KB) [file 334_2023_932_MOESM2_ESM.docx]

**Electronic Supplementary Material 2**

Phytolith assemblages reflect variability in human land use and the modern environment

N.H.Witteveen, C. White, B.A. Sanchez Martinez, R. Booij, A. Philip, W.D. Gosling, M.B. Bush, C.N.H. McMichael

Vegetation History and Archaeobotany

*Corresponding author:*

*Department of Ecosystem and Landscape Dynamics, Institute for Biodiversity and Ecosystem Dynamics, University of Amsterdam, Science Park 904, 1098 GE Amsterdam, Netherlands*

*Email:* [*n.h.witteveen@uva.nl*](mailto:n.h.witteveen@uva.nl)

*Description of modern lifestyle of Saramaccan Maroons*

Today, Saramacca Maroons depend on the forest for fishing, hunting for bushmeat, and gathering (Price, 2011). Palms such as *Astrocaryum sciophilum* (Bugru maka)*, Euterpe oleracea* (Podosiri)*, Geonoma baculifera, Oenocarpus bacaba* (Kumbu*)* and *Attalea maripa* (Maripa) are used for food, oil, and construction (van Andel and Ruysschaert, 2014). Trees such as *Ceiba pentandra* (Kankan)*, Eperua falcata* (Walaba)*, Inga* spp., *Spondias mombin* (Mope)*, Loncocarpus* spp. and *Dicorynia* *guianensis* (Basralocus) are used for timber, food and other purposes (van Andel and Ruysschaert, 2014). On small plantations of ±0.5 ha (maximum 6 ha), which have been deforested by fire and logging, shifting cultivation of *Manihot esculenta* (Cassava) is practiced. Also, *Ipomoea batatas* (Sweet potato), *Saccharum* *officinarum* (Sugarcane), Alocasia/Colocasia (Tayer), *Zea* *mays L.* (Corn), *Oryza* *sativa,* and *Oryza* *globerrima* (Rice and African rice) and *Arachis* *hypogea* (Peanuts) are planted (Price, 1991). The small plantations are surrounded by forests and secondary vegetation quickly takes over when plots are often left fallow (Hoffman, 2009).

The shifting cultivation usually has a specific order of crops. First, men cut and burn the forested vegetation to create space for the plantation, afterwards women plant crops. First *Saccharum* *officinarum* (Sugarcane), *Zea* *mays L.* (Corn), Musaceae (Banana/Bakove) and *Ipomoea batatas* (Sweet potato) are planted. On the 12^th^ or 15^th^ of March, *Oryza* (Rice) is planted, and afterwards Alocasia/Colocasia (Tayer) is planted. Cecropiaceae and *Heliconia* species grow in or near plantations as weeds.


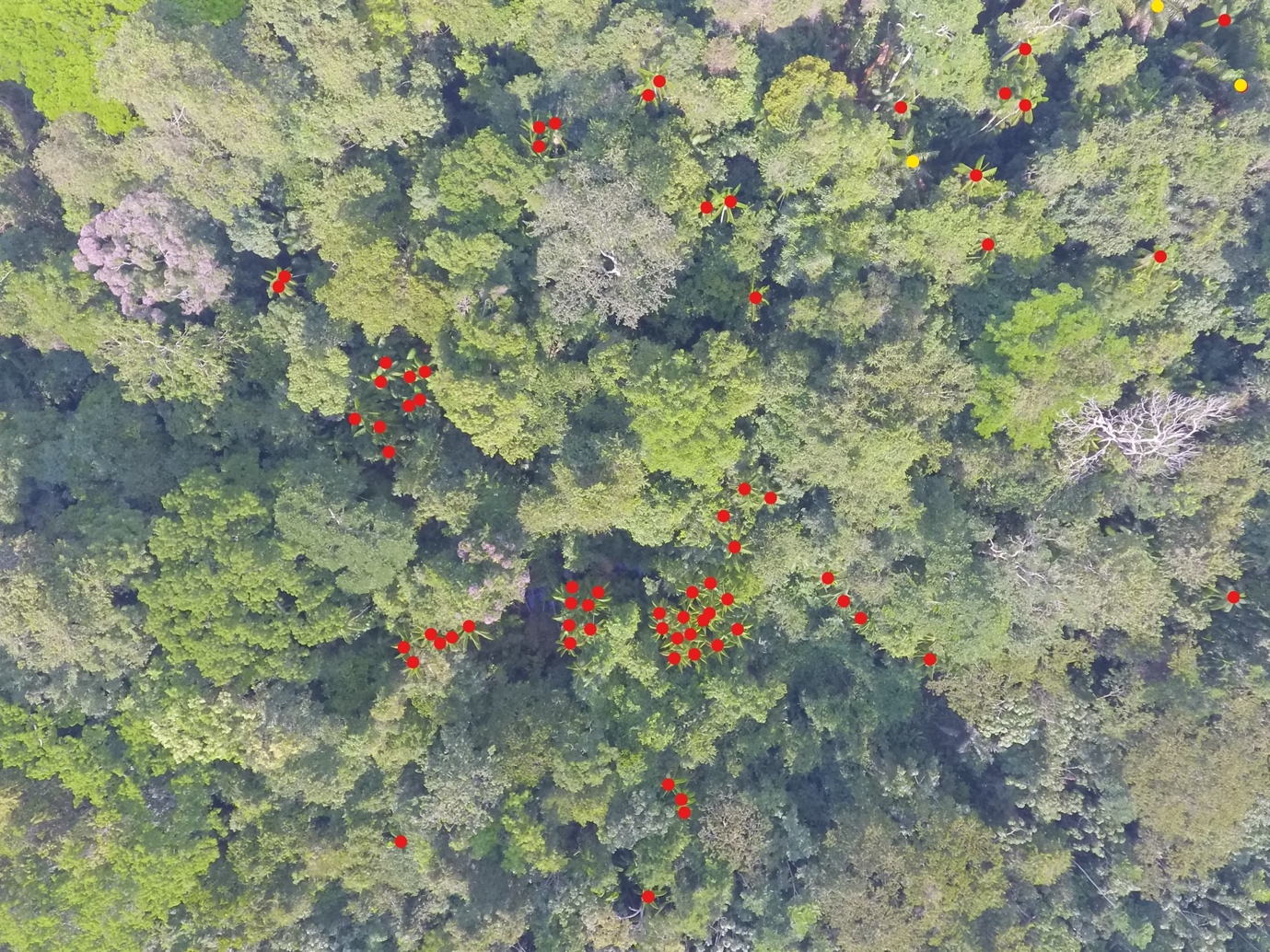


**ESM 2 Fig. 1** Palms tagged from UAV image, *E. oleracea* in red and other palms in yellow.


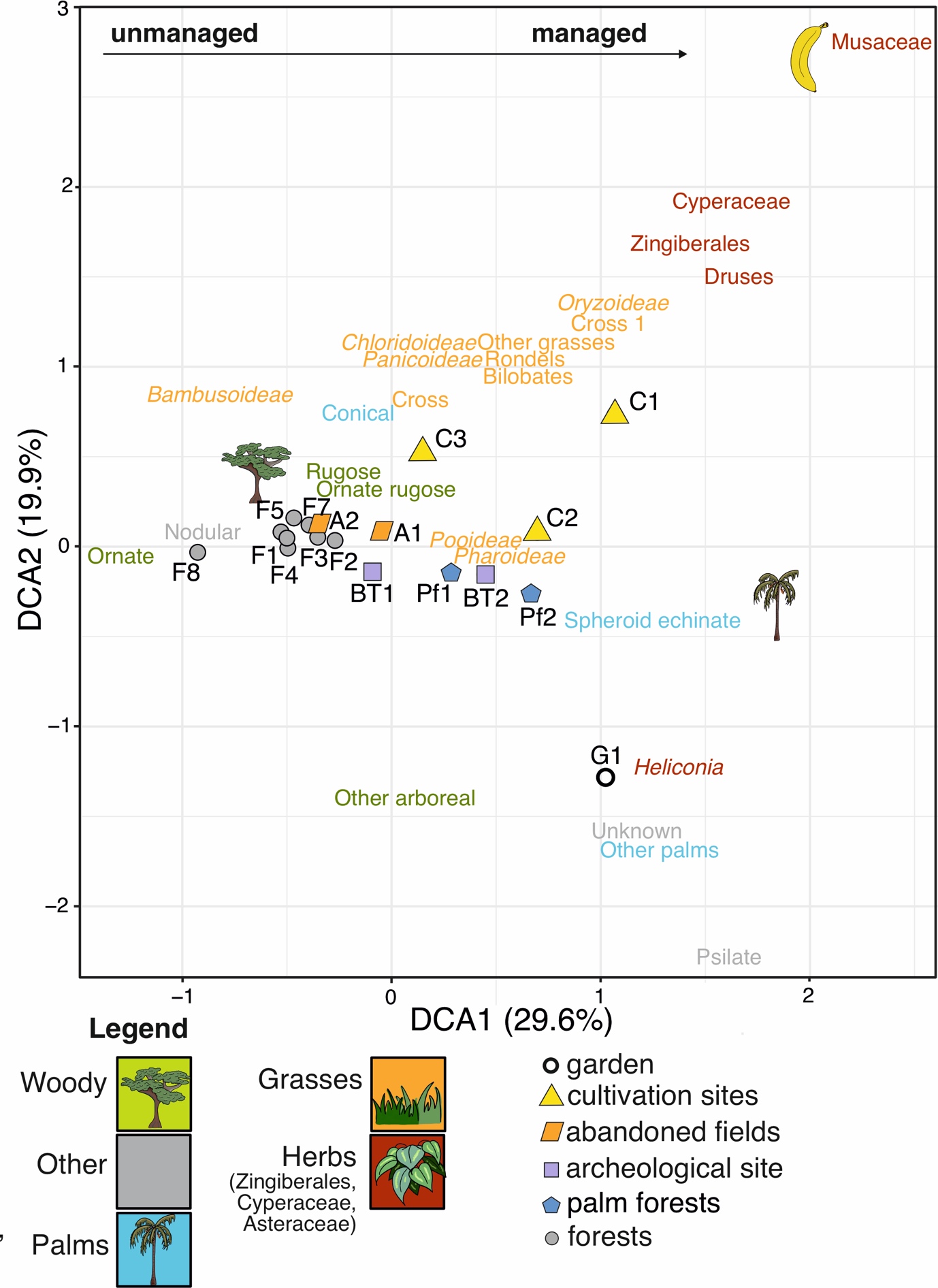


**ESM 2 Fig. 2** Detrended Correspondence Analysis (DCA) of phytolith percentage data from all the sampled sites, with DCA1 explaining 29.6% of the variance and DCA2 explaining 19.9%. Sites are color and symbol coded (see legend), phytolith morphotypes are color coded and icons represent the types of plants associated with the phytolith morphotypes
